# Decontaminating eukaryotic genome assemblies with machine learning

**DOI:** 10.1186/s12859-017-1941-0

**Published:** 2017-12-01

**Authors:** Janna L. Fierst, Duncan A. Murdock

**Affiliations:** Department of Biological Sciences, University of Alabama, Tuscaloosa, 35487 AL USA

**Keywords:** DNA sequencing, High-throughput, Genome assembly, Contamination, Sequence filtering

## Abstract

**Background:**

High-throughput sequencing has made it theoretically possible to obtain high-quality *de novo* assembled genome sequences but in practice DNA extracts are often contaminated with sequences from other organisms. Currently, there are few existing methods for rigorously decontaminating eukaryotic assemblies. Those that do exist filter sequences based on nucleotide similarity to contaminants and risk eliminating sequences from the target organism.

**Results:**

We introduce a novel application of an established machine learning method, a decision tree, that can rigorously classify sequences. The major strength of the decision tree is that it can take any measured feature as input and does not require a priori identification of significant descriptors. We use the decision tree to classify *de novo* assembled sequences and compare the method to published protocols.

**Conclusions:**

A decision tree performs better than existing methods when classifying sequences in eukaryotic *de novo* assemblies. It is efficient, readily implemented, and accurately identifies target and contaminant sequences. Importantly, a decision tree can be used to classify sequences according to measured descriptors and has potentially many uses in distilling biological datasets.

**Electronic supplementary material:**

The online version of this article (doi:10.1186/s12859-017-1941-0) contains supplementary material, which is available to authorized users.

## Background

Low-cost DNA sequencing, computing power and sophisticated assembly algorithms have made it possible to readily assemble genome sequences. However, most organisms do not live in sterile environments and extracted DNA may be contaminated with foreign DNA from associated microbiota [1–3] and endosymbionts [4]. Laboratory reagents and procedures can also introduce foreign DNA [5–7] and eliminating these sequences remains a challenge [8]. Contaminants end up sequenced and assembled along with the DNA of the target organism and, if not eliminated, will become part of the assembled genome sequence.

Contamination errors are frequent in public databases [9–11]. For example, Merchant et al. [10] identified microbial contamination in genome sequences of the cattle *Bos taurus* and an additional 50% of the publicly available genomes they analyzed. Contamination has also been reported in human [7, 12] and microbiome [6] sequences. Crisp et al. [11] analyzed horizontal gene transfer (HGT) in 40 metazoan genomes but excluded 9 from HGT analyses due to extensive contamination.

Contamination can mislead scientific studies. For example, contaminant sequences may be mistaken for HGT or complicate efforts to analyze HGT. In the Crisp study discussed above [11] genes initially classified as the result of HGT but later marked as probable contaminants had common characteristics. Sixty-nine of the nematode *Caenorhabditis japonica* HGT-derived genes were not physically linked to metazoan genes, lacked introns and were likely contaminants. A separate study [9] reported that several genes in the nematode *C. angaria* genome sequence were thought to be HGT-derived but analyses revealed 14% of the assembled genome was contributed by bacterial contaminants. Analyses of the sea anemone *Nematostella vectensis* genome [13] indicated a shikimic acid pathway not previously found in metazoans [14] but a later study found these genes were from proteobacteria ‘consorts’ and not the result of HGT [4]. The tardigrade *Hypsibius dujardini* genome was reported as 17% HGT-derived [15] but later analyses indicated large scale [16–18] contamination and an actual HGT-derived content of 1-2% [3].

Current decontamination methods eliminate known or well-characterized contaminants. For example, the software package DeconSeq filters sequences based on a contaminant database [19]. However, contaminants in *de novo* assembly projects are often not known. In this situation filtering methods must eliminate sequences based on nucleotide similarity to possible contaminants [20] or select target sequences based on similarity to known sequences in public databases [21]. Both of these approaches risk eliminating DNA from the target organism. For example, for eukaryotic genomes possible contaminants include large segments of bacteria, plants, fungi, viruses and archaea. The sheer number of possible sequences leaves filtering methods prone to ‘overfitting’ a model of contaminant identity as sequences from the target organism may resemble contaminants due to random chance. This is especially problematic when working with sequences from non-model organisms as there may be few representatives in public databases.

Conversely, sequences from the target organism may resemble contaminants because they result from true HGT. Aggressively eliminating these sequences can remove true HGT. For example, pre-assembly filtering for possible contaminants removed horizontally-transferred *Wolbachia* sequences from the first version of the *Drosophila ananassae* genome sequence [22]. Subsequent analysis and re-assembly revealed that > 1 Mb of the *Wolbachia* genome had been transferred into *D. ananassae* [22, 23].

Here, we introduce a novel application of a supervised machine learning method, a decision tree, for identifying target and contaminant DNA in *de novo* genome assembly projects. Supervised machine learning works by constructing a model from a set of training data and using this model to predict classification responses. Decision trees do not require data transformations or normalizations and produce simple, easily interpretable relationships. Their simplicity means they are well-suited for classifying data with straightforward but nonlinear relationships to predictors. Decision trees are well-established in machine learning but not commonly used in biology or bioinformatics.

**Fig. 1:**
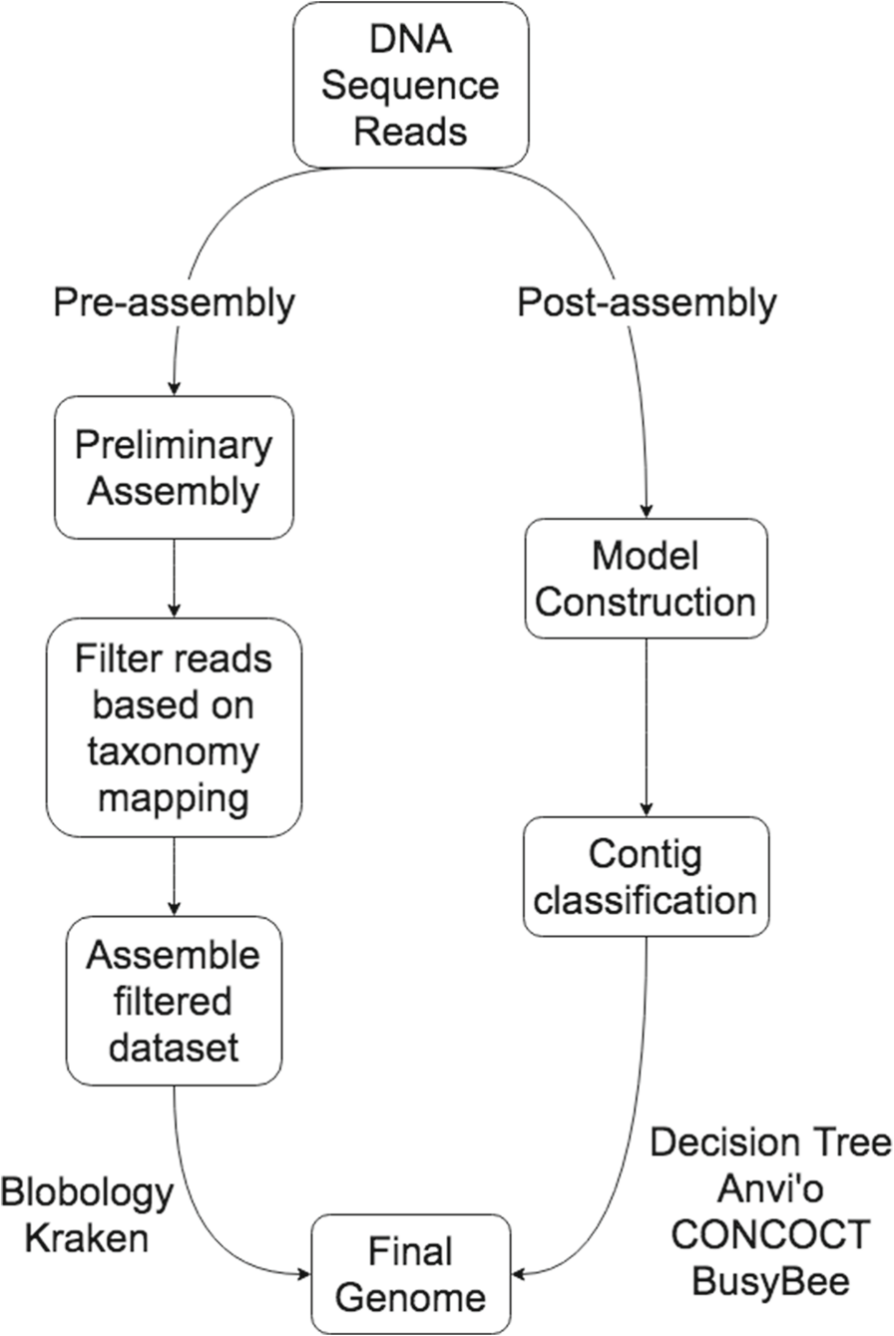
The workflow from raw DNA sequence reads to assembled genome sequence for Anvi’o with CONCOCT binning, Busybee, Blobology, Kraken, and the decision tree. Both Blobology and Kraken required pre-assembly, filtering for target and contaminant reads, and final assembly. The decision tree, Anvi’o and Busybee filtered for target and contaminant scaffolds by constructing models and classifying contiguous sequences after assembly

The majority of sequence filtering approaches have been developed for metagenomic datasets and an important question is whether methods developed for ‘binning’ microbial species can be co-opted for decontaminating eukaryotic genome sequences. For example, the frequency of short DNA ‘words’ of length *k* or *k*-mers can be used to classify microbes in metagenomic datasets [24–27]. Unsupervised classification methods bin samples based on sequence feature analysis (for example, [28, 29]) or combine sequence analysis with information on DNA sequencing coverage [30], taxonomy [31], and sequence composition [32]. Additionally, there are methods that employ both unsupervised and supervised methods to bin samples (for example, [33, 34]). Here, we evaluate the performance of our decision tree methods compared to the metagenomic classification software packages Anvi’o [32] (with CONCOCT [30] binning), Busybee [34] and Kraken [20] and the sequence filtering method Blobology [21] (Fig. 1).

We found that the decision tree accurately classified target and contaminant sequences based on measured descriptors. Importantly, the decision tree did not require a priori identification of significant descriptors and identified informative measures in constructing the model. Current decontamination methods can be time-consuming and require multiple manual steps that reduce reproducibility. In contrast, decision tree decontamination is readily implemented. The generality of the method means there are potentially many uses in biology.

## Results

### Genome sequences

We implemented our decision tree on three empirical datasets and twenty simulated datasets. The ‘real’ organisms included two nematodes from laboratory cultures, *C. remanei* and *C. latens*, that were found to be contaminated with microbes and one rotifer, *Adineta vaga*. The bdelloid rotifer *A. vaga* is both ameiotic and asexual and 8% of its genes are of non-metazoan origin [35]. We included *A. vaga* to determine if a decision tree could accurately separate foreign DNA from horizontally transferred DNA in an organism with high levels of confirmed HGT [35–37]. In order to test the methods on a range of genome-contaminant data structures we also simulated genomic and transcriptomic libraries from the published gene sequences of the plant *Arabidopsis thaliana*, the nematode *C. elegans*, the fruitfly *D. melanogaster*, and the pufferfish *Takifugu rubripes*. We contaminated each of these with a single microbe, the yeast *Candida albicans*, a low coverage mix of the microbial species listed above, an archaeon from the microbial dark matter project [38] and a mix of *Homo sapiens* and the common microbial contaminant *Bradyrhizobium sp.* [5, 6].

### Prokaryotic contaminants in empirical genome sequences

**Fig. 2:**
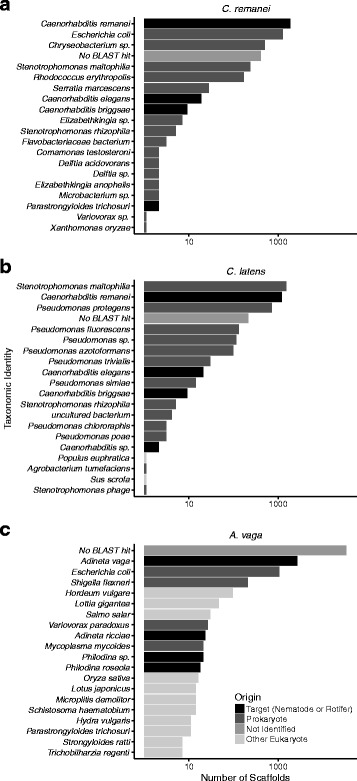
The top 20 organisms identified in BLAST analysis of the empirical genome sequences for (**a**) *C. remanei* (**b**) *C. latens* (**c**) *A. vaga*. For *C. remanei* the most common BLAST hit was *C. remanei*, followed by two likely contaminants and scaffolds that could not be assigned origin with BLAST. For *C. latens* the most common BLAST hit was the microbial contaminant *S. matophilia* followed by *C. remanei*, a second contaminant *P. protegens*, and scaffolds that could not be assigned origin. For *A. vaga* the majority of scaffolds could not be assigned origin with BLAST, likely due to the low number of rotifer sequences in public databases

The *C. remanei* genome sequence was estimated to be 131 Mb (Table 1) by flow-cytometry [39, 40] and initial analyses with Basic Local Alignment Search Tool (BLAST) [41] indicated that the assembly contained excess sequence due to microbial contaminants. The most prevalent taxonomic origin in the entire assembled genome set was *C. remanei* (Fig. 2) and the second most prevalent origin was the microbial contaminant *E. coli*. The third most prevalent organism was an unnamed *Chryseobacterium species*, also a microbial contaminant. 409 scaffolds could not be assigned taxonomic origin with BLAST.

**Table 1:** Estimated genome sizes and published assembly sizes for organisms used in this study

Organism	Estimated size (Mb)	Assembled sequence (Mb)
*C. remanei*	131 [39, 40]	118.36 [52]
*C. latens*	131	122.22
*A. vaga*	244 [63]	218.07 [35]
*A. thaliana*	125 [64]	135.67 [65]
*C. elegans*	100 [66]	103.02 [67]
*D. melanogaster*	175 [68]	142.57 [69]
*T. rubripes*	390 [70]	393.31 [50]
*A. radiobacter*	7.27	7.27 [71]
*C. albicans*	14.86	14.85 [72]
*E. coli*	4.64	4.64 [73]
*P. aeruginosa*	6.27	6.27 [49]
*Ralstonia sp.*	5.25	5.25

Empirical study organisms are listed in the upper portion, simulated target organisms are listed in the center portion and simulated contaminants are listed in the lower portion of the table. There is no published estimate of genome size for *C. latens* and we used the genome size of the closely related [42] *C. remanei* as an estimated *C. latens* genome size

For *C. latens* the most prevalent taxonomic origin was a microbial contaminant, *Stenotrophomonas maltophilia*, that was also found in the *C. remanei* assembled sequence (Fig. 2). The second most prevalent taxonomic origin was *C. remanei*. This is likely because *C. latens* is a recently described species (previously *C.* species 23 [42]) and there are few *C. latens* sequences in public databases. *C. remanei* and *C. latens* are closely related and partially interfertile [43]. We were not able to identify a taxonomic origin for 429 of the assembled scaffolds.

For the *A. vaga* dataset there were non-metazoan BLAST alignments as expected under a model of high HGT. BLAST could not identify a taxonomic origin for 34,264 *A. vaga* scaffolds (Fig. 2) which was likely due to the low number of rotifer sequences in public databases. In order to identify probable contaminants we focused on an unusual pattern of 989 BLAST alignments to a single strain of *E. coli* (K-12 strain C3026), 206 BLAST alignments to a single strain of the human pathogen *Shigella flexneri* (4c), and 26 BLAST alignments to the microbe *Variovorax paradoxus*.

### Identifying contaminants with predictor variables

We removed the target species genome sequences from the NCBI nucleotide (nt/nr) database and used BLAST to assign taxonomic origin. We aligned DNA and RNA sequence reads to each genome and calculated 8 predictor variables for scaffolds: (1) length, (2) GC content, (3) mean DNA sequencing coverage, (4) mean RNA sequencing coverage, (5) percent of scaffold covered in DNA alignment, (6) percent of scaffold covered in RNA alignment, (7) GC content of aligned DNA reads, and (8) GC content of aligned RNA reads.

**Fig. 3:**
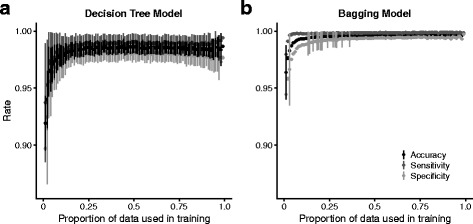
Accuracy, sensitivity and specificity for (**a**) decision tree and (**b**) bagging decision tree models. Decision tree models achieved high accuracy, sensitivity and specificity but were influenced by variation in the training dataset. The bagging decision tree model achieves high accuracy, sensitivity and specificity with lower variance between models constructed with different training datasets. For the decision tree models accuracy, sensitivity and specificity plateau with >25% of the data used in training while the performance of the bagging model plateaus with >40% of the data used in training

**Fig. 4:**
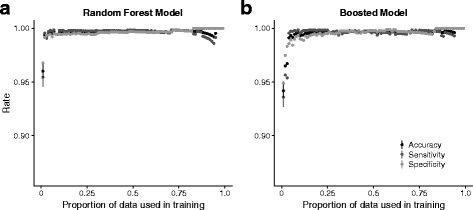
Accuracy, sensitivity and specificity for (**a**) random forest and (**b**) boosted decision tree models. Both random forest and boosted decision tree models resulted in high accuracy, sensitivity and specificity but showed non-monotonic responses to the training datasets

We selected a portion of the scaffolds with BLAST-assigned taxonomy as a training set and used the remainder of scaffolds with BLAST-assigned taxonomy as a test dataset. We used the training set to construct a decision tree and used this tree to classify each of the test scaffolds as either target or contaminant. We varied the portion of the dataset used in training from 1-99% and calculated the mean and standard deviation of accuracy, sensitivity, and specificity across 100 replicates (results for *C. remanei* Fig. 3a). Here, model error was the percent of scaffolds in the test dataset that had a BLAST-assigned origin and were mis-classified. Accuracy was measured as 1-error. Sensitivity was calculated as *T* *P*/(*T* *P*+*F* *N*) where *TP* was the number of true positives and *FN* was the number of false negatives. Specificity was calculated as *T* *N*/(*T* *N*+*F* *P*) where *TN* was the number of true negatives and *FP* was the number of false positives. True positives were correctly identified target organism sequences and true negatives were correctly identified contaminants. Accuracy, sensitivity, and specificity plateaued with >40% of the data used for training (Figs. 3 and 4) and we used 50% of the dataset for decision tree training.

**Fig. 5:**
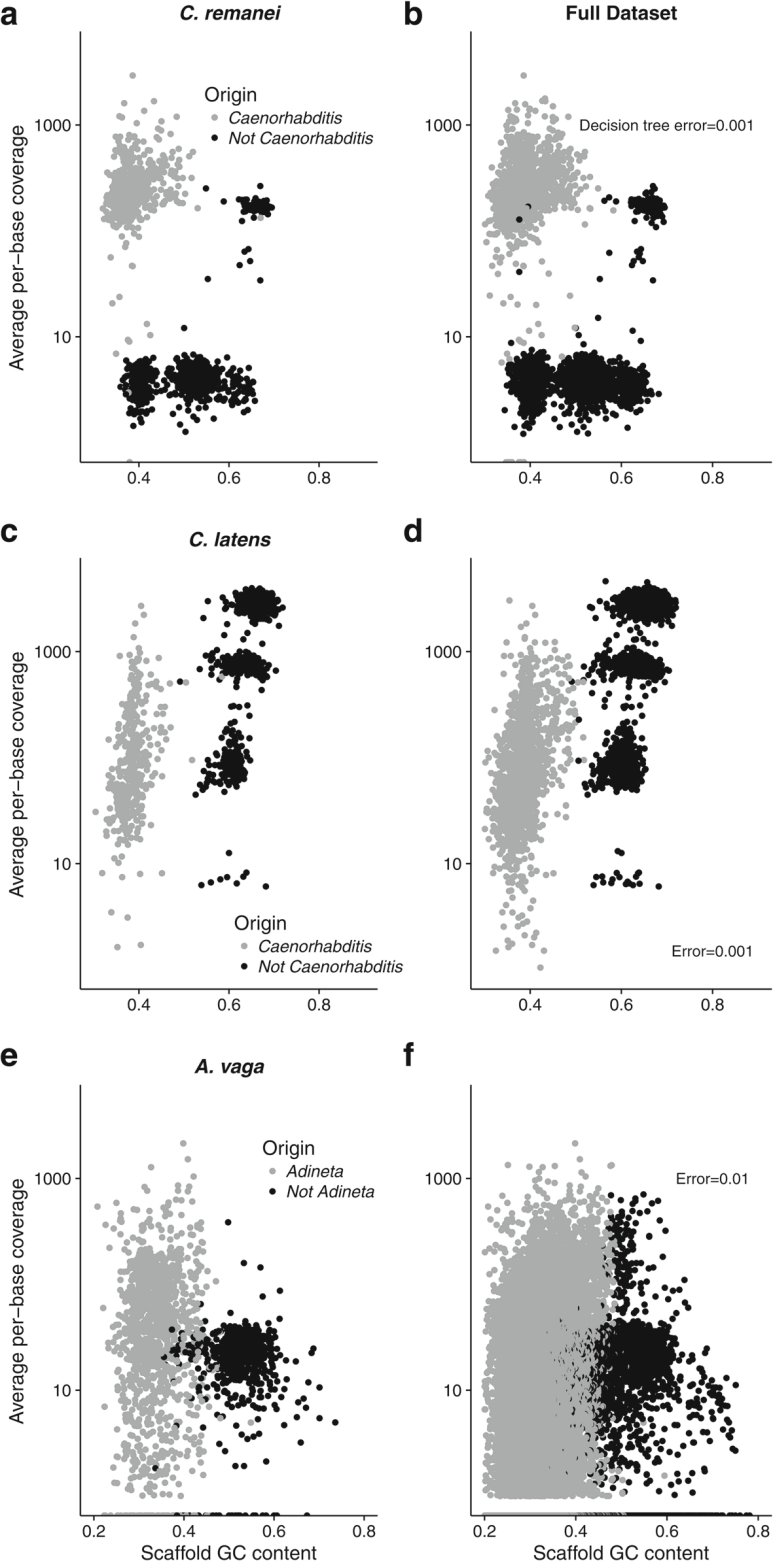
GC content and the average per-base sequencing coverage for individual scaffolds in the empirical datasets (**a**) *C. remanei* training; (**b**) *C. remanei* full dataset; (**c**) *C. latens* training; (**d**) *C. latens* full dataset; (**e**) *A. vaga* training; and (**f**) *A. vaga* full dataset. Training datasets with BLAST-identified origins are shown on the left and decision tree bagging model predictions for full datasets are shown on the right with model error

Decision trees are susceptible to bias and variance due to variation in the training dataset (Fig. 3a). In order to construct more accurate models we used a variation of a bootstrap procedure, bootstrap aggregation or ‘bagging’, that reduces the variance of the decision tree model (Fig. 3b). We also estimated the performance of random forest models (Fig. 4a) and boosted decision tree models (Fig. 4b). Accuracy, sensitivity and specificity were >99.5% for each of these models but the random forest and boosted models did not show monotonic responses to the proportion of data used in training and we used bagged decision tree models for the remainder of the analyses. Sensitivity exceeded specificity for all models (Figs. 4 and 5).

For *C. remanei* the bagging model predicted 19.38 Mb contained in 2470 scaffolds did not have a *Caenorhabditis* origin (Table 2; Fig. 5a-b). The contaminant sequences predominantly had low sequencing coverage (on average less than 10x; Fig. 5b) and GC content ranging from 35–70% or moderate sequencing coverage (on average, similar to that for scaffolds of *Caenorhabditis* origin) with high GC content (greater than 60%) although >50 scaffolds had GC/coverage profiles that deviated from this pattern. Of the 409 scaffolds without taxonomic origin the bagged decision tree model predicted 213 were contaminants.

**Table 2:** Assembled genome size and number of scaffolds before and after bagging decision tree decontamination for the empirical genome sequences

Organism	Contaminated assembly size (Mb)	Number of scaffolds	Decontaminated assembly size (Mb)	Number of scaffolds
*C. remanei*	137.74	4,566	118.36	2,096
*C. latens*	139.27	4,559	122.22	1,664
*A. vaga*	218.07	38,875	217.44	35,988

For *C. latens* 17.06 Mb contained in 2896 scaffolds were of non-*Caenorhaditis* origin (Table 2; Fig. 5c-d). The model predicted that 28 of the 429 scaffolds without BLAST-identified origin were contaminants. The contaminant scaffolds had moderate-to-high sequencing coverage that actually exceeded the sequencing coverage of the *C. latens* scaffolds for roughly 1/3 of the contaminant scaffolds. The GC content of contaminant scaffolds was 55-70% while the GC content of the *C. latens* scaffolds was 30-50%.

The decision tree predicted 0.62 Mb contained in 2887 scaffolds were contaminants in the *A. vaga* genome sequence (Table 2; Fig. 5e-f). The model predicted 1593 of the 34,262 scaffolds without BLAST-identified taxonomy were contaminants. The contaminant scaffolds were small sequences with a median size of 59 bp and a mean size of 169 bp. In contrast, the true *Adineta* scaffolds had a median size of 408 bp and a mean size of 1080 bp. Contaminant scaffolds had GC content >40% while the *Adineta* scaffolds had GC content <45%.

### Predictor variables

**Fig. 6:**
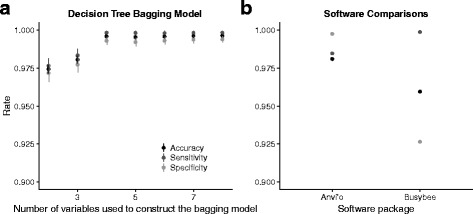
Accuracy, sensitivity and specificity for (**a**) the decision tree bagging model constructed with 2-8 predictors and (**b**) Anvi’o with CONCOCT binning and Busybee. Acccuracy and sensitivity for the decision tree bagging model plateau with 4 predictors but small increases in specificity resulted from additional predictors. Anvi’o had the highest specificity compared to the decision tree bagging model or Busybee while Busybee had the highest sensitivity

For each dataset we randomly selected 50% of the scaffolds with BLAST-assigned taxonomy as a training dataset and constructed bagged decision tree models for 2-8 variables. We repeated this procedure 1000 times and calculated the mean and standard deviation of accuracy, sensitivity, and specificity for each of these predictor combinations. Here, we focus on results for *C. remanei* (Fig. 6a). Mean DNA sequencing coverage and mean RNA sequencing coverage had the highest Gini importances and a model constructed solely with these predictors was able to correctly classify >97% of the *C. remanei* dataset. When a third predictor, the percent of the scaffold covered in RNA alignment, was added the model correctly classified >98% of the dataset. Model accuracy and sensitivity plateaued above 99.5% when a fourth variable, scaffold GC content, was included but specificity increased slightly as successive predictors were added to the model.

### Software comparisons

We compared the decision tree bagging model results against those produced by Anvi’o [32] with CONCOCT binning [30], Busybee [34], Kraken [20] and Blobology [21]. Processing our sequencing files with Anvi’o was time-intensive and because of that we chose to proceed with the default setting and analyzed the 2304 scaffolds >2500 bp. We calculated accuracy, sensitivity and specificity based on this smaller scaffold set. Anvi’o [32] separated the contaminated *C. remanei* genome sequences into 18 bins however 3 of these contained only 1 scaffold. Seven bins contained primarily *C. remanei* sequences. Specificity was high (Fig. 6b) and Anvi’o misclassified just 2 *Chryseobacterium* scaffolds as *Caenorhabditis*. However, the overall Anvi’o accuracy rate was lower at 98.1% with 5 misclassified scaffolds and 38 scaffolds that were entirely unclassified. Of these, 21 were *Caenorhabditis* sequences and sensitivity was 98.5%.

**Fig. 7:**
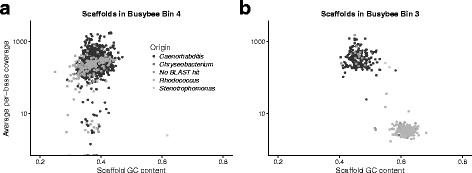
Busybee bin 4 (**a**) contained primarily scaffolds of *Caenorhabditis* or unknown origin with few microbial contaminants while Busybee bin 3 (**b**) was a hetereogeneous mix of sequences with different origins. The scaffolds in Busybee bin 3 separated by taxonomic origin when visualized by scaffold GC content and sequencing coverage

Busybee [34] separated the contaminated *C. remanei* genome sequences into 5 bins. Busybee had a sensitivity rate of 99.89% (Fig. 6b) and placed just 2 *Caenorhabditis* scaffolds in microbial bins but the 2 *Caenorhabditis* bins (Fig. 7) contained 166 microbial scaffolds. Busybee bin 4 contained the majority of the *C. remanei* scaffolds with few microbial scaffolds (Fig. 7a) but Busybee bin 3 was a heterogeneous mix of scaffolds from *C. remanei* and *Rhodococcus* species (Fig. 7b).

Pre-assembly filtering methods can not be evaluated with accuracy, sensitivity and specificity and instead we measured the resulting genome size and genic completeness with BUSCO [44] and CEGMA [45]. BUSCO searches for a set of 982 orthologous genes thought to exist in single-copy in metazoans and CEGMA searches for a set of 248 ultra-conserved eukaryotic orthologous genes. For *C. remanei* the Blobology protocol [21] resulted in a genome sequence 0.75 Mb smaller than the decision tree genome sequence. We repeated the Blobology protocol focusing on a single contaminant order, *Xanthomonadales*, and assembled a complete genome sequence for the microbe *S. maltophilia* [46]. Using Kraken [20] for pre-assembly filtering resulted in a genome sequence 9.3 Mb shorter than the decision tree sequence. The decision tree assembled sequence contained a greater proportion of the BUSCO and CEGMA gene sets when compared with Blobology and Kraken (Table 3).

**Table 3:** Percentage of orthologous genes found by BUSCO and CEGMA in the *C. remanei* genome sequences

Protocol	BUSCO	CEGMA complete form	CEGMA partial form
Decision tree	99.59%	94.35%	98.79%
Blobology	98.98%	94.35%	97.18%
Kraken	89.82%	84.68%	88.31%

There were 982 genes in the BUSCO nematode set and 248 ultra-conserved eukaryotic genes in the CEGMA set

### Identifying contaminant sequences in simulated genomes

We assembled the simulated libraries with low coverage microbial sequences, archaeons, and *H. sapiens*/*Bradyrhizobium* contaminants but BLAST failed to identify any scaffolds with these taxonomic origins in the resulting genome sequences. Accordingly, we focused on the simulated libraries with microbial and fungal contaminants for decision tree decontamination.

**Fig. 8:**
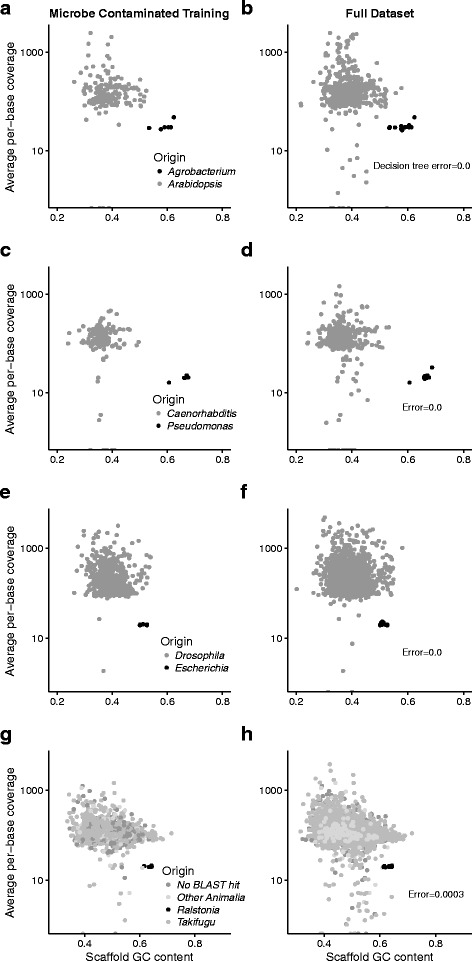
GC content and average per-base sequencing coverage for the simulated datasets contaminated with microbial DNA. Training datasets are shown on the left and bagging decision tree predictions are shown on the right for **a**-**b**) *A. thaliana*; **c**-**d**) *C. elegans*; **e**-**f**) *D. melanogaster*; and **g**-**h**) *T. rubripes*. The microbial genomes were GC-rich relative to the target organisms and a simple decision tree based on GC content and sequencing coverage predicted scaffold origin with low error for each dataset

**Fig. 9:**
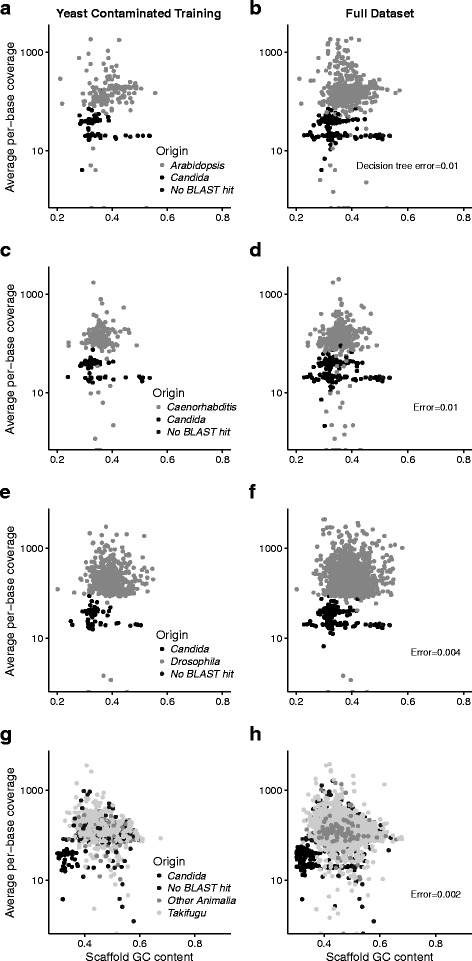
GC content and average per-base sequencing coverage for the simulated datasets contaminated with *C. albicans* DNA. Training datasets and bagging decision tree predictions are shown for **a**-**b**) *A. thaliana*; **c**-**d**) *C. elegans*; **e**-**f**) *D. melanogaster*; and **g**-**h**) *T. rubripes*. *C. albicans* and the target organisms had similar GC contents and the bagging decision tree predictions were based on a complex relationship that included multiple predictors and mRNA data

The simulated libraries with microbial contaminants were disentangled with decision tree models constructed solely on the scaffold GC content and the average per-base DNA sequencing coverage (Fig. 8). The simulated microbial contaminants had scaffold GC contents of 50-69% while the target organisms had scaffold GC contents of 24-72%. The GC content of the assembled *C. albicans* scaffolds ranged from 23-53% and was similar to the target organisms which had GC contents of 24-72% (Fig. 9). Accordingly, the *C. albicans*-contaminated simulated libraries showed poor discrimination with a decision tree model constructed with scaffold GC content and average per-base sequencing coverage (error rates >10%). For each simulated library contaminated with *C. albicans* we constructed a model with the full eight variables to increase prediction accuracy > 99% (Fig. 9).

## Discussion

We have developed a novel implementation of a decision tree, an established machine learning method, for distilling and decontaminating *de novo* assembled genome sequences. Our method filters based on any measurable characteristic. Here, we have focused on eight predictors and constructed decision tree models for empirical and simulated datasets. These models accurately predicted target or contaminant status for >99% of the scaffolds for which we could assign taxonomic origin with BLAST [41]. Importantly, we were able to classify scaffolds as target or contaminant in the absence of BLAST information based on predictor variables. Decision tree decontamination works on measurable sequence characteristics and is particularly useful for non-model organisms and those with low representation in public databases. Additionally, the influence of existing contamination in public databases can be limited by reducing training dataset size and manually curating training data.

### Decontamination and dataset GC structure

In our model runs the complexity of the decision tree was influenced by the GC structure of the target and contaminant genome sequences. For example, the simulated datasets with bacterial contaminants were accurately decontaminated with a simple model based on scaffold GC content and average per-base sequencing coverage. Although genomic GC content varies broadly, metazoan genomes skew towards an enrichment of AT nucleotides while the GC content of bacterial genomes ranges from <15% to >75% [47]. In the simulated libraries these differences, coupled with discrete differences in the average per-base sequencing coverage, were large enough to accurately discriminate between target and contaminant sequences. These results indicate that discriminating between target and contaminant sequences in empirical datasets may be straightforward if the target and contaminant genomes have very different GC structures. For example, an easily discriminated case may be identifying sequences from a single high-GC contaminant in an invertebrate genome assembly.

The simulated libraries were created from high-quality genome sequences assembled with high certainty. Despite this, there was large variability in the estimated sequencing coverage (for example, Fig. 8). The ART [48] simulation software we used produces sequence reads according to a model based on real Illumina datasets and includes coverage variability and substitution, insertion and deletion errors. However, very large coverage values like the maximum sequencing coverage estimates we have reported here result in part from difficulties that arise in aligning relatively short 150 bp sequence reads to long repeats and other complex structures in metazoan genome sequences. Even in these ‘ideal’ simulated situations, the average per-base sequencing coverage did not reliably separate target and contaminant DNA sequences.

### Including multiple predictor variables.

For our empirical datasets we were able to classify targets and contaminant sequences with relatively high accuracy (>90%) with decision tree models constructed solely on GC content and DNA sequencing coverage. However, achieving >99% accuracy, sensitivity and specificity required decision tree models constructed with at least 4 predictor variables. This was also true for the simulated datasets contaminated with the yeast *C. albicans* (Fig. 9).

The eight predictor variables we chose reflected different aspects of the assembly process and the biological basis of the sequences. For example, scaffold length is a measure of how reliably that segment of DNA assembled and depends on the sequencing coverage in the total sequence read library and the complexity of the sequence. In contrast, the percent of the scaffold covered in aligning RNA reads to the assembled scaffold measures gene density which varies by taxonomic origin. For example, the microbial *P. aeruginosa* genome is 89.4% coding sequence [49] while the genome of the vertebrate *T. rubripes* is 22.23% coding sequence [50]. Each of these descriptors had different Gini importances for the decision tree models constructed in this study and the minimal sets of predictor variables would likely differ for different target-contaminant combinations.

### Software comparisons

We compared the *C. remanei* assembled sequences produced by our decision tree model with those produced by Anvi’o with CONCOCT binning [30, 32], Busybee [34], Blobology [21] and Kraken [20]. Both Anvi’o with CONCOCT binning and Busybee were developed for disentangling metagenomic datasets and microbial sequences. Anvi’o excelled at identifying microbial sequences but the decision tree model had higher accuracy and sensitivity than Anvi’o when constructed with >3 predictor variables. Busybee had an extremely high sensitivity but lower accuracy and specificity relative to both the decision tree models and Anvi’o. Metagenomic tools that are tuned for success with microbial sequences may not be capable of achieving the same levels of success in decontaminating eukaryotic datasets.

Anvi’o was time-intensive in pre-processing and analyzing sequences and could not accurately classify scaffolds < 2500 bp. In comparison, Busybee [34] was readily available through a web portal and accurately classified >95% of the complete *C. remanei* scaffold set in less than 10 minutes. The time-limiting step in decision tree decontamination is aligning the DNA and RNA reads to the assembled genome. Once this is complete model construction and scaffold selection can be completed within minutes.

Kraken overfit a model of contaminant identity and the assembled *C. remanei* sequence was shorter and had the smallest complement of orthologous genes [44] when compared with either the decision tree or Blobology sequences. Blobology resulted in an assembled sequence that was 99% of the length of the decision tree assembled sequence. The decision tree sequence had a greater percentage of orthologous genes [44, 45] when compared with both the Blobology and Kraken sequence. These results indicate that decontaminating and producing a complete assembled genome may require tools that are specifically tuned for certain scenarios. For example, the *C. remanei* sequence contained at least one full bacterial genome [46]. The raw ALLPATHS-LG [51] assembly contained many partial bacterial sequences but Blobology’s targeted filtering and re-assembly produced a high-quality sequence for the contaminant *Stenotrophomonas maltophilia*.

### Classification errors

We began this study motivated by the goal of decontaminating assembled genome sequences without removing HGT. Under this conceptual framework we might expect that classification errors relate to horizontally transferred sequences but we did not find this. Each method produced a small number of errors and we were able to investigate these individually. For example, 2 scaffolds in the *C. remanei* dataset were predicted as contaminants by the decision tree model but were identified as related to the nematode *Parastronglyoides* by BLAST. Although *Parastronglyoides* is a nematode, the full list of BLAST hits for these sequences included multiple microbes and no other nematodes. One scaffold was binned with *E. coli* sequences by both Anvi’o [32] and Busybee [34] and although BLAST identified it as related to the nematode *C. elegans*, the full list of BLAST hits for this sequence included multiple microbes as well. The BLAST-assigned taxonomic identity for some of these errors may reflect contamination in public databases.

Other mis-classifications did not reflect contamination errors. Anvi’o [32] failed to classify two scaffolds that did not express mRNA but that BLAST identified as closely related to *C. elegans*. These scaffolds contained gene sequences encoding *srh*-266, a serpentine or chemoreceptor, and a glycosyltransferase with conserved single copy homologs across *Caenorhabditis*, *Drosophila* and *Danio rerio*. Busybee [34] binned several megabases of *Rhodococcus* sequence and a *Rhodococcus* plasmid with *Caenorhabditis* sequences including a 137,654 bp scaffold that expressed mRNA and contained a gene sequence encoding a homolog to *C. elegans* fibrillin-1, an extracellular matrix protein with a human homolog that results in Marfans syndrome when mutated.

These sequences did not have any readily observable patterns and were likely binned together because of similarities in *k*-mer frequencies. We do not understand how mutation and functional convergence influence the evolution of genome sequences. Complete knowledge of supervised and unsupervised sequence analysis methods will require a deeper understanding of the rules that govern change at a genomic level.

## Conclusions

Here, we have presented a novel implementation of a decision tree model for decontaminating *de novo* sequence assemblies. Our method is readily implemented, reproducible and fast. We have shown that it can rigorously decontaminate sequences and is useful for non-model organisms. Machine learning methods are established in other disciplines but not yet common in biology. We hope that this example demonstrates the utility of machine learning methods for distilling meaning in biological datasets.

## Methods

### Empirical genome sequences

Genomic DNA was isolated from *C. remanei* and *C. latens* nematode worms (for detailed experimental methods, see [52]). We sequenced one overlapping paired-end genomic library with an average fragment size of 180bp (as required by the assembly software ALLPATHS-LG [51]) for each nematode. We sequenced three mate pair genomic libraries with average fragment sizes of 0.7-2, 2-4, and 4-7 kb for *C. remanei* and three mate pair libraries with average fragment sizes of 4-6 kb, 6-9 kb and 9-12 kb for *C. latens*. Libraries were sequenced as 2 x 101nt reads with an Illumina HiSeq. We sequenced these libraries at high depth and in order to avoid biased errors we used the *k*-mer filter protocol in the software package Stacks [53] to pre-filter the overlapping paired end fragments by *k*-mer frequency spectra. We used *k*=15 and removed reads with greater than 12 rare *k*-mers (single occurrences) and greater than 51 abundant *k*-mers (here, defined as 20,000 or more occurrences). We used the ALLPATHS-LG (version 52488) [51] genome assembly software package which performs *k*-mer spectra correction of sequencing errors (with *k*=25), builds contiguous sequences with a de Bruijn graph from the 180bp reads, and constructs scaffolds with mate pair libraries.

We isolated total RNA from mixed-stage populations of *C. remanei* and *C. latens*, purified mRNA, synthesized cDNA libraries and sequenced these libraries as 2 x 101nt reads with an Illumina HiSeq. We used the MAKER2 software package [54] to annotate protein-coding genes (for detailed methods see [52]).

The *A. vaga* sequence read libraries were obtained from the Sequence Read Archive [55] and assembled genome sequences were obtained from the National Center for Biotechnology Information (NCBI) [56]. For details on DNA and RNA isolation, sequencing, assembly and annotation see [35].

Sequencing the *C. remanei* paired-end genomic library produced 367,673,013 overlapping pairs (statistics for *C. remanei* libraries are given in Additional file 1: Table S2). We removed 75,850,115 sequence reads with rare *k*-mers and 22,433,210 sequence reads with abundant *k*-mers resulting in 637,062,701 retained reads. Sequencing the *C. latens* paired-end library produced 171,027,578 overlapping pairs (*C. latens* library statistics are given in Additional file 1: Table S3). We removed 46,491,040 reads with rare *k*-mers and 11,359,209 reads with abundant *k*-mers resulting in 284,204,907 retained sequence reads. We filtered the sequenced mRNA libraries for adapter contamination and retained 26,170,962 *C. remanei* reads and 32,459,744 *C. latens* reads for transcript assembly. Statistics for the *A. vaga* libraries used in this study are given in Additional file 1: Table S4 and statistics for the simulated genomic and transcriptomic libraries are given in Additional file 1: Tables S5 and S6.

### Simulated genome sequences

We simulated DNA sequence reads for *A. thaliana*, *C. elegans*, *D. melanogaster* and *T. rubripes* and contaminated these libraries with sequences from *Agrobacterium radiobacter*, *Pseudomonas aeruginosa*, *Escherichia coli*, *Ralstonia sp.* 5_7_47FAA [6], *C. albicans*, microbial dark matter archaea [38], *Homo sapiens* and the common microbial contaminant *B. sp.* BTAi1 [5, 6]. Due to the large size of the human genome [57] we simulated sequences from the *H. sapiens* mitochondrial chromosome and chromosome X, Y, IV, XII, and XX. GenBank accessions for genome sequences are listed in Additional file 1: Table S1.

We simulated Illumina sequence read datasets with the software package ART (version ART-MountRainier-2016-06-05) [48]. We generated two genomic DNA libraries for each target organism, one a 150 bp paired-end library with a 270 bp fragment, standard deviation of 30 bp (resulting in, on average, 10% overlap between the paired reads) and average per-base sequencing coverage of 100x and one a 150 bp mate pair library with a 2500 bp fragment, standard deviation of 50 bp and average per-base sequencing coverage of 33x. We also generated two genomic DNA libraries for each contaminant organism with the parameters listed above but lower average per-base sequencing coverage. For the work reported here, the contaminant paired-end sequencing coverage was 20x and the mate pair sequencing coverage was 10x. We combined target and contaminant to create contaminated libraries and assembled genome sequences with ALLPATHS-LG [51].

We also simulated RNA libraries with the software package ART [48]. For each target organism we simulated a 100bp paired-end library with a 400bp fragment, standard deviation of 50 bp and an average sequence coverage of 30x. For each contaminant organism we simulated a library with the parameters listed above but a lower sequencing coverage of 10x.

### Assigning taxonomic identity to scaffolds

We used the Basic Local Alignment Search Tool (BLAST 2.3.1+) to identify the single best BLASTn [41] match (expect threshold=10; word size=28; match/ mismatch scores=1,-2, gap costs=linear) for each assembled scaffold. We used the NCBI nt database and for each target organism we removed that species genome sequences from the database. For the *C. remanei* and *C. latens* datasets we filtered these by genus and assigned each scaffold a taxonomic identity of ‘*Caenorhabditis*’, not ‘*Caenorhabditis*’ or ‘No BLAST hit.’ Note that some of the scaffolds identified in this way may actually be matching residual contamination in other *Caenorhabditis* assemblies (for example, the suspected contaminants in *C. angaria* [9] and *C. japonica* [11]). This may contribute to error in our datasets but we chose to maintain consistency with published results.

BLAST could not assign taxonomic identity for 34,264 *A. vaga* scaffolds (88% of the total scaffolds; 35.5 Mb of assembled sequence; 16.3% of the genome) as expected for a non-model organism. We assigned the 34,264 unidentifiable scaffolds a taxonomic identity ‘No BLAST hit,’ the *E. coli* K-12 strain C3026, *S. flexneri* 4c and *V. paradoxus* scaffolds a taxonomic identity of ‘Not *Adineta*’ and the remaining scaffolds a taxonomic identity of ‘*Adineta*.’

We classified the simulated assembled scaffolds by genus with BLAST with the exception of the *T. rubripes* datasets. Each of these contained scaffolds that BLAST could not assign taxonomic identity to and scaffolds that aligned to other metazoans. In order to focus on contaminants we assigned each scaffold a taxonomic identity of ‘No BLAST hit,’ ‘Other Animalia,’ ‘*Takifugu*,’ or the identified contaminant.

### Calculating descriptors for training scaffolds

We used the program GMAP-GSNAP (version 2017-03-17) [58] to align DNA and RNA sequence reads to each assembled genome sequence. Reads were mapped as paired, correct orientation was required and we selected the single highest scoring location (no multimapping was allowed). Mate pair libraries may contain chimeric sequences and other artifacts that would align incorrectly and we eliminated these from coverage calculations. We used the BBMap/BBTools software package (version 37.32) [59] to calculate the length and GC content of each assembled scaffold. We also calculated the average sequencing fold coverage across each scaffold, the percent of scaffold covered in aligning reads to the assembled sequence and the GC content of the reads aligned to the assembled sequence for both DNA and RNA libraries.

### The decision tree algorithm

The decision tree framework measured the information gain contributed by each variable according to the entropy *H*(*S*)=−*Σ*_*x*∈*X*_ *p*_*x*_ *l* *o* *g*_2_ *p*_*x*_ where *H*(*S*) was the entropy of dataset *S*, *X* were the classes in *S* and *p*(*x*) was the probability of *x* or the proportion of *x* in the dataset. Information gain was computed as *I* *G*(*A*,*S*)=*H*(*S*)−*Σ*_*t*∈*T*_ *p*(*t*)*H*(*t*) where *I* *G*(*A*,*S*) was the difference in entropy from splitting on the variable or attribute *A*, *T* were subsets of *S* and *p*(*t*) was the proportion of *t*. Importantly, the decision tree framework did not require assumptions regarding which variables were significant and could classify data based on any measurable feature.

Data were classed according to Gini’s diversity index where $G = \Sigma _{i}^{n_{c}} p_{i} (1- p_{i})$. Here, *n*_*c*_ was the number of classes and and *p*(*i*) was the observed fraction of class *i* observations in the set. Training data was used to identify useful descriptors and construct a binary decision tree according to $$ \widehat{y}=\underset{y=1,\dots, K}{\arg\ \min }{\varSigma}_{k=1}^K\widehat{P}\left(k|x\right)C\left(y|k\right). $$ Here, $\hat {y}$ was predicted classification, *K* the number of classes, $\hat {P}(k|x)$ posterior probabilities, *C*(*y*|*k*) the cost of misclassification and arg min the input that minimized the value of the function.

We constructed bootstrapped aggregated or ‘bagged’ decision tree models by generating B different bootstrapped training datasets and averaging the predictions to obtain $\hat {f}_{bag}(x) = \frac {1} {B} \sum ^{B}_{b=1}\hat {f}^{*b}(x)\phantom {\dot {i}\!}$ where $\hat {f}^{*b}(x)$ is the set of predictions obtained from the *b*th bootstrap training set. We measured the Gini importance of each variable in the model as $I = G_{parent} - G_{node_{1}} - G_{node_{2}}\phantom {\dot {i}\!}$, where *G* is the diversity index defined above. We also implemented a random forest model by bootstrapping training datasets and constructing decision trees from a random sample *m* of the *p* predictor variables. Here, *m=4* and we constructed 5000 trees per model run. A random forest model is an extension of a bagging model and the two are equivalent when *m=p*. We implemented a boosted model by fitting decision trees to the residuals of a shallow tree and summing across these trees. Here, we allowed 2 splits in each tree, used a shrinkage parameter of 0.01 and fit 10 trees per model run.

### Implementing Anvi’o with CONCOCT binning

We used the assembled genome sequence to create an Anvi’o (v2.4.0 “Pyrenees”) contig database with *k*-mer frequency (computed at *k*=4), GC content, open reading frames and predicted bacterial single-copy core genes for each scaffold [32]. We processed scaffolds > 2500 bp with anvi’o to estimate sequencing coverage profiles (mean, standard deviation and average coverage for inner quartiles) and characterize single-nucleotide variants for our DNA and RNA sequences. We used CONCOCT (v0.4.0) [30] for binning within Anvi’o. Briefly, CONCOCT bins metagenomic samples by analyzing both *k*-mer frequency and sequencing coverage across assembled scaffolds.

### Implementing Busybee

We uploaded the assembled genome sequence as a.fasta to the Busybee (version 2017-01-09) [34] web portal (https://ccb-microbe.cs.uni-saarland.de/busybee). We enabled both taxonomic and functional annotation and uploaded our set of 8 predictor variables as a custom annotation file. We set the minimum contig length at 1000 bp, the minimum contig length for border points at 1000, the minimum contig length for cluster points at 2000, the *k*-mer length at 5, the probability at 0.0, the minimum points in neighbourhood at 30, and the transformation at standard.

### Implementing Blobology

We implemented the Blobology protocol [21] to compare with decision tree decontamination. Briefly, a preliminary assembly was performed using ABySS-PE (version 1.9.0) [60]. For the *C. latens* and *C. remanei* datasets, only the standard paired-end data was assembled. The source FASTQs were aligned back to this new assembly using GMAP-GSNAP [58], and the assembly was classified using NCBI BLAST [41]. The BLAST results, assembly, and alignment were analyzed with blobtools (v0.9.19) [21]. The resulting TAGC plot was used to determine filtering conditions based on the GC content and average per-base sequencing coverage of each assembled contiguous sequence (GC<0.45 and coverage>2 for A.vaga, GC<0.45 for *C. latens*, and three passes for C. remanei [GC<0.65, GC<0.6 && coverage>11, GC<0.5 && coverage >5]). A list of contiguous sequences which met these conditions was generated, and paired-end and mate pair reads matching these contiguous sequences were selected using custom scripts alongside standard GNU tools and samtools [61]. The post-filtering reads were assembled with ABySS-PE [60] or ALLPATHS-LG [51]. Assemblies were evaluated using QUAST (version 4.5) [62].

### Implementing Kraken

We also filtered our empirical assemblies with Kraken (version 0.10.5-beta)[20] to compare with the Decision Tree. Briefly, a preliminary assembly was performed using ABySS [60], and the source FASTQs were aligned to this assembly using GMAP-GSNAP [58]. Each sequence in this assembly was classified using Kraken [20]. Contiguous sequences successfully classified by Kraken were assumed to be contaminants due to the content of Kraken’s standard database (generated from NCBI FTP on April 4, 2016). This list was used to generate a list of contiguous sequences to keep from the data, and reads matching those contiguous sequences were selected using custom scripts alongside standard GNU tools and samtools [61]. The post-filtering reads were assembled with ABySS [60] or ALLPATHS-LG [51]. We used QUAST (version 4.5) [62], BUSCO (v2.0) with the nematodea_odb9 database [44] and CEGMA (version 2.4) [45] to assess the completeness of the decision tree, Blobology, and Kraken assemblies.

## Additional file

Additional file 1 Supplement: Fierst and Murdock, Decontaminating eukaryotic genome assemblies with machine learning. (PDF 9713 kb)
