# Supplementary material for: Decontaminating eukaryotic genome assemblies with machine learning
Source: BMC Bioinformatics. 2017 Dec 1;18:533. doi: 10.1186/s12859-017-1941-0 (PMC5709863; doi:10.1186/s12859-017-1941-0)
Supplement: Additional file 1 — Supplement: Fierst and Murdock, Decontaminating eukaryotic genome assemblies with machine learning. (PDF 9713 kb) [file 12859_2017_1941_MOESM1_ESM.pdf]

Supplement: Fierst and Murdock, Decontaminating eukaryotic genome assemblies with machine learning

Supplementary Tables

Table 1 GenBank accession information for genome sequences used in this study. Empirical study organisms are listed in the upper portion, simulated target organisms are listed in the center portion and simulated contaminants are listed in the lower portion of the table.

Organism	Accession
Caenorhabditis remanei	GCA_001643735
C. latens	
Adineta vaga	GCA_000513175
Arabidopsis thaliana	GCA_000001735
C. elegans	GCA_000002985
Drosophila melanogaster	GCA_000001215
Takifugu rubripes	GCA_000180615
Agrobacterium radiobacter	GCA_000016265
Candida albicans	GCA_000182965
Escherichia coli	GCA_000005845
Pseudomonas aeruginosa	GCA_000006765
Ralstonia sp.	GCA_000165085

Table 2 C. remanei libraries used for genome assembly and protein-coding gene annotations.

	Average Insert (bp)	SD (bp)	Sequences
Paired-end	-21	20	735,346,026
Mate pair 1	910	200	47,692,376
Mate pair 2	2,310	400	45,286,070
Mate pair 3	4,810	500	23,521,698
mRNA	100	100	47,430,962

Table 3 C. latens libraries used for genome assembly and protein-coding gene annotations.

	Average Insert (bp)	SD (bp)	Sequences
Paired-end	-20	20	342,055,156
Mate pair 1	4,800	400	31,125,622
Mate pair 2	7,300	400	30,211,040
Mate pair 3	10,800	500	34,605,684
mRNA	100	100	81,531,840

Table 4 A. vaga libraries used for calculating sequencing coverage. The 'P-e 1' library was sequenced as 101bp paired-end sequences on an Illumina HiSeq 1000 machine and the 'P-e 2' library was sequenced as 160bp paired-end sequences on an Illumina Genome Analyzer IIx machine. The '454 DNA' and '454 mRNA' libraries were sequenced as single-end sequences on a 454 GS FLX Titanium instrument. The 'GAIIx mRNA' library was sequenced as 76bp single-end sequences on an Illumina Genome Analyzer IIx instrument.

	Average Insert (bp)	SD (bp)	Sequences
P-e 1	450	100	65,758,350
P-e 2	862	200	171,129,084
	Average Length (bp)	SD (bp)	Sequences
454 DNA	531	101	3,822,869
454 mRNA	585	86	1,613,684
GAIIx mRNA	76	0	17,607,592

Table 5 The number of sequence reads in simulated libraries with mRNA.

Organism	Paired-end	Mate pair	mRNA
A. thaliana	79,632,290	26,272,288	15,706,110
C. albicans	3,663,488	1,831,744	1,714,938
C. elegans	66,857,200	22,062,878	12,700,350
D. melanogaster	94,842,988	31,215,228	26,705,580
T. rupribes	228,622,576	72,057,510	25,551,884

Table 6 The number of sequence reads in simulated contaminant libraries.

Organism	Paired-end	Mate pair
A. radiobacter	969,720	484,860
E. coli	618,880	309,440
Ralstonia. sp.	695,100	347,550
P. aeruginosa	835,240	417,620
Low coverage microbial mix	10,902,314	3,632,632
Bradyrhizobium/Homo	9,657,728	4,826,110
MDM Archaeon	152,862	76,420

Table 7 Contaminated and decontaminated assembly statistics for simulated libraries.

Organism/Contaminant	Contaminated size (Mb)	Number of scaffolds	Decontaminated size (Mb)	Number of scaffolds
A. thaliana/A. radiobacter	122.62	830	115.48	816
A. thaliana/C. albicans	129.78	1019	115.43	806
C. elegans/P. aeruginosa	104.33	823	98.16	810
C. elegans/C. albicans	112.55	1026	98.24	818
D. melanogaster/E. coli	126.66	1918	122.27	1909
D. melanogaster/C. albicans	136.43	2090	122.14	1887
T. rubripes/Ralstonia sp.	331.36	7843	326.63	7833
T. rubripes/C. albicans	359.24	5396	344.83	5186

Table 8 Statistics for the empirical genome sequences assembled with the Blobology [1] and Kraken [2] protocols.

Organism	Preliminary assembly size (Mb)	Number of scaffolds	Method	Assembly size (Mb)	Number of scaffolds
C. remanei	133.47	62,404	Blobology	117.75	1,662
			Kraken	109.20	1,898
C. latens	146.37	311,657	Blobology	117.14	1,466
			Kraken	116.41	1,637
A. vaga	185.37	120,046	Blobology	166.69	93,150
			Kraken	145.21	95,421

Table 9 Percentage of orthologous genes found by BUSCO [3] and CEGMA [4] in the C. latens and A. vaga genome sequences.

Protocol	Organism	BUSCO	CEGMA complete form	CEGMA partial form
Decision tree	*C. latens*	98.07%	95.56%	98.39%
Blobology	*C. latens*	98.47%	95.56%	98.19%
Kraken	*C. latens*	94.60%	87.90%	91.94%
Decision tree	*A. vaga*	91.72%	98.39%	98.79%
Blobology	*A. vaga*	91.62%	93.95%	96.77%
Kraken	*A. vaga*	72.39%	75.81%	77.82%

Supplementary Figures

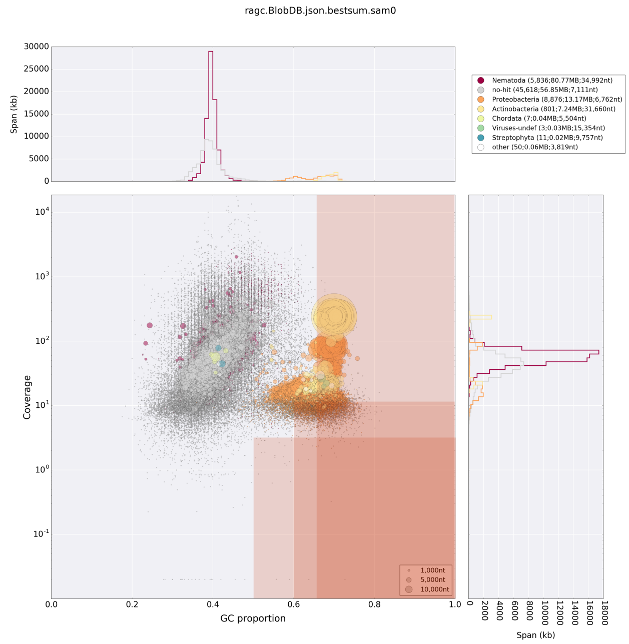

Figure 1 C. remanei blobplot. The red highlighted regions show contigs that were excluded when filtering.

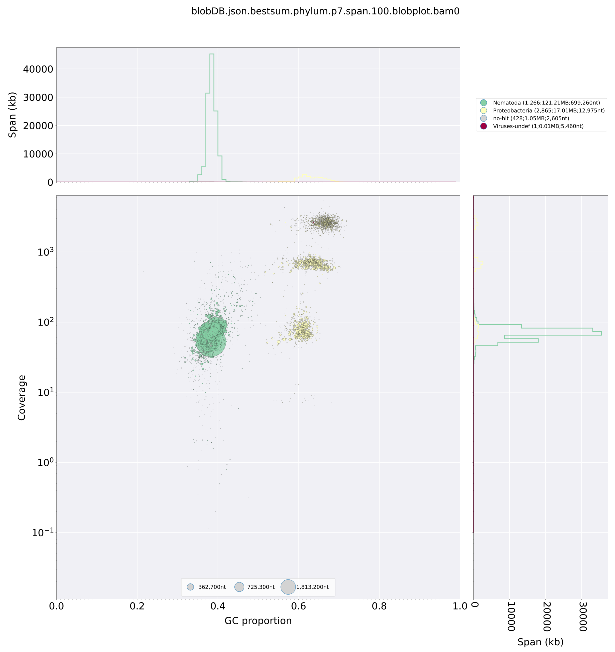

Figure 2 C. latens blobplot.

Table 10 Gini Importance Measures for the empirical datasets.

	C. remanei	*C. latens*	*A. vaga*
Scaffold length	4.61	3.31	138.57
Scaffold GC content	81.27	195.79	334.12
Average per-base DNA sequencing coverage	154.31	61.56	53.47
Average per-base RNA sequencing coverage	146.67	60.89	9.96
Percent of scaffold covered in DNA alignment	23.96	12.49	76.32
Percent of scaffold covered in RNA alignment	110.67	33.10	5.35
GC content of aligned DNA reads	77.54	184.85	223.01
GC content of aligned RNA reads	77.35	26.74	8.80

Table 11 Gini Importance Measures for the simulated libraries with microbial contaminants.

	A. thaliana	*C. elegans*	*D. melanogaster*	*T. rubripes*
Scaffold GC content	21.08	17.82	2.59	6.29
Average per-base DNA sequencing coverage	2.38	1.32	9.34	9.49

Table 12 Gini Importance Measures for the simulated libraries with C. albicans.

	A. thaliana	*C. elegans*	*D. melanogaster*	*T. rubripes*
Scaffold length	6.42	8.23	6.62	7.34
Scaffold GC content	20.89	11.60	23.68	80.40
Average per-base DNA sequencing coverage	90.20	80.62	120.10	52.09
Average per-base RNA sequencing coverage	13.89	33.11	17.96	11.92
Percent of scaffold covered in DNA alignment	5.11	6.80	3.73	6.90
Percent of scaffold covered in RNA alignment	54.56	46.99	39.10	35.46
GC content of aligned DNA reads	15.16	8.79	18.29	57.69
GC content of aligned RNA reads	20.84	22.25	13.10	9.09

Author details

References

1. Kumar S, Jones M, Koutsovoulos G, Clarke M, Blaxter M. Blobology: exploring raw genome data for contaminants, symbionts, and parasites using taxon-annotated GC-coverage plots. Frontiers in Genetics. 2013;4(237):1–12.
2. Wood DE, Salzberg SL. Kraken: ultrafast metagenomic sequence classification using exact alignments. Genome Biology. 2014;15:R46.
3. Simao FA, Waterhouse RM, Ioannidis P, Kriventseva EV, Zdobnov EM. BUSCO: assessing genome assembly and annotation completeness with single-copy orthologs. Bioinformatics. 2015;31(19):3210–3212.
4. Parra G, Bradnam K, Korf I. CEGMA: a pipeline to accurately annotate core genes in eukaryotic genomes. Bioinformatics. 2007 May;23(9):1061–1067.

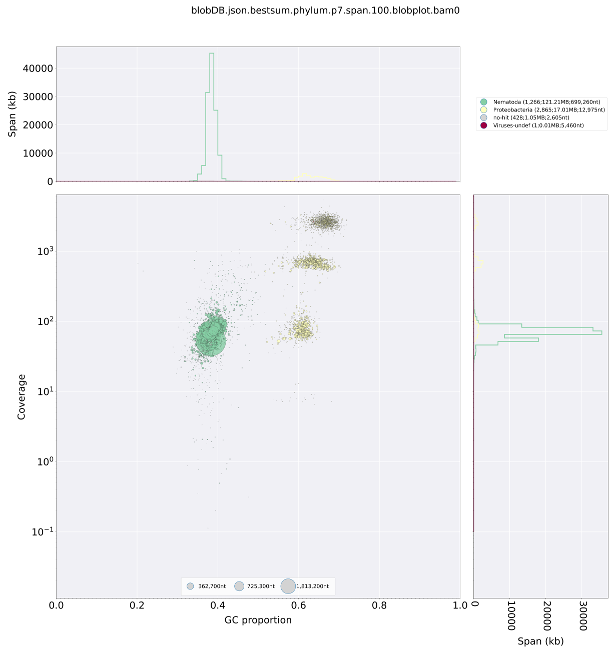

Figure 3 A. vaga blobplot.
